# In Silico Bioinformatics Followed by Molecular Validation Using Archival FFPE Tissue Biopsies Identifies a Panel of Transcripts Associated with Severe Asthma and Lung Cancer

**DOI:** 10.3390/cancers14071663

**Published:** 2022-03-25

**Authors:** Laila Salameh, Poorna Manasa Bhamidimarri, Narjes Saheb Sharif-Askari, Youssef Dairi, Sarah Musa Hammoudeh, Amena Mahdami, Mouza Alsharhan, Syed Hammad Tirmazy, Surendra Singh Rawat, Hauke Busch, Qutayba Hamid, Saba Al Heialy, Rifat Hamoudi, Bassam Mahboub

**Affiliations:** 1 Sharjah Institute for Medical Research, College of Medicine, University of Sharjah, Sharjah 27272, United Arab Emirates; lailasalameh80@gmail.com (L.S.); pbhamidimarri@sharjah.ac.ae (P.M.B.); nsharifaskari@sharjah.ac.ae (N.S.S.-A.); sara-hammoudeh@hotmail.com (S.M.H.); amahdami@sharjah.ac.ae (A.M.); qalheialy@sharjah.ac.ae (Q.H.); 2 Dubai Health Authority, Dubai 4545, United Arab Emirates; ydairy@hotmail.com (Y.D.); maalsharhan@dha.gov.ae (M.A.); hamadtirmazy@yahoo.com (S.H.T.); drbassam_mahboub@yahoo.com (B.M.); 3 Collage of Medicine, Mohammed Bin Rashid University of Medicine and Health Sciences, Dubai 505055, United Arab Emirates; ssrawat26july@gmail.com (S.S.R.); saba.alheialy@mbru.ac.ae (S.A.H.); 4 Lübeck Institute of Experimental Dermatology, University of Lübeck, Lübeck 23562, Germany; hauke.busch@uni-luebeck.de; 5 Meakins-Christie Laboratories, Research Institute of the McGill University Healthy Center, Faculty of Medicine, Montreal, QC H3A 0G4, Canada; 6 Division of Surgery and Interventional Science, University College London, London NW3 2QG, UK

**Keywords:** asthma, lung cancer, GSEA analysis, bioinformatics, *POSTN*, *LUM*, *BCL3*

## Abstract

**Simple Summary:**

The present study identified a panel of transcripts involved in the pathogenesis of both severe asthma and lung cancer. The genes identified using publicly available transcriptomics data were validated on cell lines, plasma samples, and archival tissue biopsies from asthmatic and lung cancer patients. The functional roles of the identified markers in both the diseases were ascertained from the literature. These molecular markers might be useful for diagnosing lung cancer at early stages.

**Abstract:**

Severe asthma and lung cancer are both heterogeneous pathological diseases affecting the lung tissue. Whilst there are a few studies that suggest an association between asthma and lung cancer, to the best of our knowledge, this is the first study to identify common genes involved in both severe asthma and lung cancer. Publicly available transcriptomic data for 23 epithelial brushings from severe asthmatics and 55 samples of formalin-fixed paraffin-embedded (FFPE) lung cancer tissue at relatively early stages were analyzed by absolute gene set enrichment analysis (GSEA) in comparison to 37 healthy bronchial tissue samples. The key pathways enriched in asthmatic patients included adhesion, extracellular matrix, and epithelial cell proliferation, which contribute to tissue remodeling. In the lung cancer dataset, the main pathways identified were receptor tyrosine kinase signaling, wound healing, and growth factor response, representing the early cancer pathways. Analysis of the enriched genes derived from the pathway analysis identified seven genes expressed in both the asthma and lung cancer sets: *BCL3*, *POSTN*, *PPARD*, *STAT1*, *MYC*, *CD44*, and *FOSB*. The differential expression of these genes was validated in vitro in the cell lines retrieved from different lung cancer and severe asthma patients using real-time PCR. The effect of the expression of the seven genes identified in the study on the overall survival of lung cancer patients (*n* = 1925) was assessed using a Kaplan–Meier plot. In vivo validation performed in the archival biopsies obtained from patients diagnosed with both the disease conditions provided interesting insights into the pathogenesis of severe asthma and lung cancer, as indicated by the differential expression pattern of the seven transcripts in the mixed group as compared to the asthmatics and lung cancer samples alone.

## 1. Introduction

Lung cancer is a heterogeneous disease associated with a poor prognosis and is a leading cause of cancer mortality in both men and women [1]. It is expected that, by 2035, around 3 million deaths worldwide will be attributed to lung cancer [2]. The pathophysiology of lung cancer is very complex and vaguely understood. Studies to date show that repeated exposure to carcinogens, especially cigarette smoke, leads to dysplasia of the lung epithelium, and continuous exposure may lead to specific gene mutations that disrupt the cell cycle, thereby promoting carcinogenesis. The most common genetic mutations identified in small-cell lung cancer (SCLC) patients are in the genes *MYC*, *BCL2*, and *p53*, whereas mutations in genes such as *EGFR*, *KRAS*, and *p16* are known to be associated with non-small-cell lung cancer (NSCLC) [3,4,5].

Like lung cancer, asthma is a heterogeneous disease of the airways, the susceptibility to which is determined by genetic characteristics and environmental triggers such as respiratory infections and cigarette smoke [6]. The heterogeneity in asthma is due to the complex mechanisms, mediators, and triggers of each phenotype. Among the subtypes of asthma, type-2-driven asthma is classified into Th2-high and Th2-low asthma. Th2-high asthma has three specific differentially expressed transcriptomic profiles, namely, periostin (*POSTN*), chloride channel regulator 1 (*CLCA1*), and serpin peptidase inhibitor, clade B, member 2 (*SERPINB2*), among which *POSTN* was found to be a more reliable surrogate marker for Th2-high asthma [7,8].

Previous studies have focused largely on the genetic alterations in asthma [9] and lung cancer [10] separately; however, to our knowledge, there are no studies that have identified the common genetic variations associated with both lung cancer and asthma. Symptoms of asthma can also be observed in other lung diseases such as chronic obstructive pulmonary disease (COPD), emphysema, and chronic bronchitis. Moreover, similar symptoms were observed in patients with early signs of lung cancer [11]. Asthmatic patients and the physicians who treat them may attribute these symptoms to uncontrolled asthma and delay treatment. Another reason for the delay could be the non-inclusion of asthmatic patients who do not smoke in the high-risk group for eligibility for lung cancer screening [12]. Hence, the early diagnosis of lung cancer is compromised.

Both intrinsic and extrinsic cellular factors can drive chronic inflammation in the bronchial epithelium, promoting lung carcinoma via specific genes. Particularly in the case of asthmatics, studies have shown that the inflammatory state may predispose to cancer in the lung and other organs such as the breast, colon, and prostate. A meta-analysis conducted in 2017 showed a significant association of asthma with lung cancer risk [13]. A single-center study conducted by us at Rashid Hospital, Dubai, demonstrated that asthmatic patients had a higher risk of developing tumors related to the breast, colon, lung, and prostate. Lung cancer in asthmatics had the longest diagnosis period (36.6 years), while prostate cancer had the shortest (16.5 years) [14].

Analysis of the mutational DNA status and the RNA and protein expression levels of specific genes can provide important information; however, the redundancy of gene function and the complexity of molecular pathways have led researchers to shift toward transcriptomic analysis. Transcriptomics has provided an exceptional opportunity to study the functional implications of genetic variability. However, to date, no molecular studies have been able to identify markers correlating with the early onset of lung cancer. With the availability of microarrays and sequencing technologies, combined with the accessibility of publicly available genomic and transcriptomic data, a multilevel in silico approach has proven to be profitable in both cancer and asthma gene investigation and pathway analysis [15]. Pathway and network analyses allow researchers to gain an understanding of the functional biology underpinning tumors, allowing a comprehensive gene list to be shortened and clustered with the identification of significant targets [16].

Hence, in this study, we aimed to identify common gene signatures differentially expressed between asthma and lung cancer, using publicly available transcriptomic datasets, and to validate the identified genes using tissue biopsies obtained from asthma and lung cancer patients by RT-qPCR. The present study may aid in the identification of biomarkers for early lung cancer detection. Consequently, this approach could facilitate the development of a comprehensive transcriptomics database that can link and predict early lung cancer susceptibility or the early stages of the development of chronic inflammation in the bronchial epithelium that could lead to lung carcinoma.

## 2. Materials and Methods

### 2.1. Study Design

#### 2.1.1. Microarray Data Selection 

The Gene Expression Omnibus (GEO) was manually mined for publicly available severe asthma and lung cancer transcriptomic data (https://www.ncbi.nlm.nih.gov/geo/, last access date was 30 June 2021) up to the end of 2020 in order to choose the appropriate microarray datasets based on inclusion and exclusion criteria. The selection criteria include studies exclusively for Homo sapiens, Caucasian, and based on a similar platform; also, studies with severe asthma cases recruited as per asthma guidelines were only considered. In the case of lung cancer, studies with non-small cell lung cancer cases were considered. Datasets were also chosen based on the tissue samples; for example, bronchial central epithelial biopsies or bronchial epithelial brush for severe asthma samples and FFPE biopsies for lung cancer were only selected. Studies based on late-stage cancer or metastasis or recurrence or involved in pharmacological manipulations were excluded. The two datasets GSE64913 and GSE29013 were selected, which met all the criteria stated above and were run on the same Affymetrix Platform U133 Plus 2.0, GPL570. 

#### 2.1.2. Patient Cohort for In Silico Analysis

A total of 17 asthmatics and 23 non-asthmatic healthy volunteers were considered from the dataset GSE64913. The transcriptomic data for these samples were obtained from epithelial brushings from both central and peripheral airways [17]. The severe asthmatics in the set were characterized by a history of at least two exacerbations in the previous year of collection, and 44% had a history of hospital admission for acute severe asthma in the preceding year of sample collection. The mean age of the subjects was 41; all the subjects were nonsmokers, except for three ex-smokers with a pack-year history <2 who had ceased smoking for at least 1 year (Table 1).

The dataset GSE29013 comprises data for formalin-fixed paraffin-embedded (FFPE) tumors from 55 patients at stages 1–3 (24 patients = stage 1; 14 patients = stage 2; 17 patients = stage 3) of non-small-cell lung carcinoma (NSCLC) [18]. The mean age of the subjects was 63.5 years; 38 of them were male; all were nonsmokers except for two (Table 1). From each FFPE block, at least 50 mm^2^ of tumor tissue was collected, and RNA extraction was performed using a patented process developed by Response Genetics Inc. (Los Angeles, CA, USA, United States Patent Application 20090092979).

#### 2.1.3. In Vivo Validation

##### Ethical Consideration

The in vivo validation in the present study was based on samples obtained from a single center; Rashid Hospital, Dubai. The study protocol was reviewed and approved by the Dubai Scientific Research Ethical Committee (DSREC) Dubai Health Authority with the ethical approval number DSREC-SR-03L2019_01.

##### Formalin-Fixed Paraffin-Embedded Tissue Samples for In Vivo Validation

**Figure 1:**
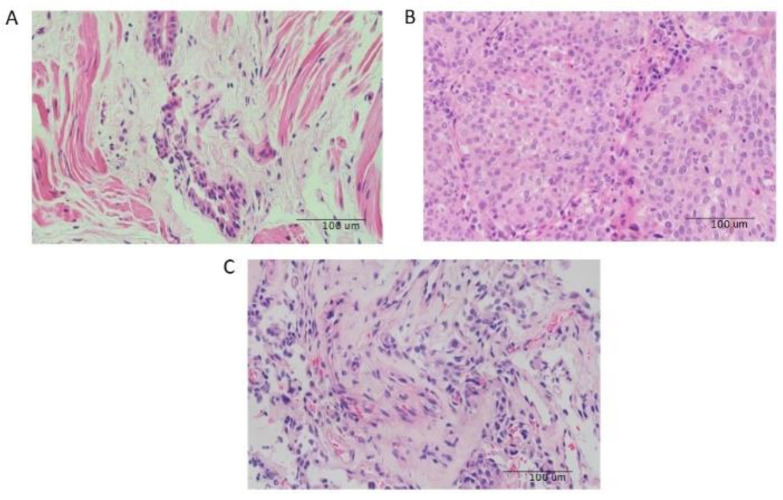
Images of slides with H&E staining for tissue sections from (**A**) asthmatic, (**B**) lung cancer, and (**C**) mixed cases.

In total, 11 biopsies from the clinical archives of Rashid Hospital, Dubai (single center), were identified that were related to the present study. The tissue samples obtained from an independent clinical cohort of four severe asthmatics who fit the criteria mentioned for severe asthma (all nonsmokers), four patients with NSCLC (three smokers), and three asthmatic patients who developed lung cancer (all nonsmokers) were used for validation by RT-qPCR (Table 2). The FFPE slides were reviewed by three pathologists (M.A., R.H., and Q.H.). The H&E slides showed the pathology of the asthmatic patients, suggesting hyperinflamed lung tissue with inflammatory regions, with a thickness of the subepithelial basement membrane indicative of tissue remodeling. However, lung cancer H&E staining (Figure 1B) showed more invasive carcinoma within the lung tissue. Interestingly, the H&E slides of asthma patients with lung cancer (Figure 1A,C) showed mixed features between asthma and lung cancer, with more adenocarcinomas in situ (AIS) of the lung, which are akin to preinvasive lesions.

The small sample size is due to the fact that, in general, it is not routine clinical practice to perform biopsies for severe asthmatics.

##### Blood Samples

Whole blood was collected from three lung cancer and three severe asthma patients in EDTA-treated tubes. The characteristics of the patients are provided in Table 3. The plasma was immediately isolated and stored in aliquots at −80 °C until further use. Total RNA was extracted from ~300 µL of plasma using the TRIzol (Invitrogen, Carlsbad, CA, USA) method.

##### Survival Analysis

An independent cohort of 1925 NSCLC patients was used to analyze the overall survival and clinical implications of the expression of the seven genes using Kaplan–Meier Plotter (https://kmplot.com/analysis/index.php?p=service&cancer=lung last access date was 28 January 2022) [19]. The details regarding age, sex, smoking history, histology, stage, grade were collected from different data sets previously published [18,19,20,21,22,23,24,25,26,27,28]. The mean age for the cohort was 64 ± 10, 58% were male, while 17.8% never smoked. Supplementary Data S1 summarizes the clinical properties for the cohort.

#### 2.1.4. In Vitro Validation

##### Cell Culture

Asthmatic cells were obtained from bronchial biopsies of the severe asthma patients, as listed in Table 4. Diseased human bronchial epithelial cells, asthmatic (DHBE-As) (Lonza, Switzerland), and lung cancer cells with different stages of non-small-cell lung carcinoma (AddexBio, San Diego, CA, USA) were commercially obtained. Details for the cell types are provided in Table 4.

A549, CALU-3, and SKLU-1 cells were grown in RPMI-1640 medium supplemented with 10% fetal bovine serum (FBS), while the asthmatic cells (DHBE, S13, and S14) were cultured in PneumaCult™ medium as described by the manufacturer. All the cells were grown in a humidified chamber at 37 °C with 5% CO_2_. The medium was changed every 2 days until the cells in the flasks were 95% confluent. The cells were then collected for RNA extraction. In total, three asthmatic and three lung cancer cell lines were used for molecular validation.

### 2.2. In Silico Analysis

#### 2.2.1. Microarray Data Analysis to Identify Differentially Expressed Genes between Severe Asthmatics and Healthy Controls in Bronchial Epithelium

**Figure 2:**
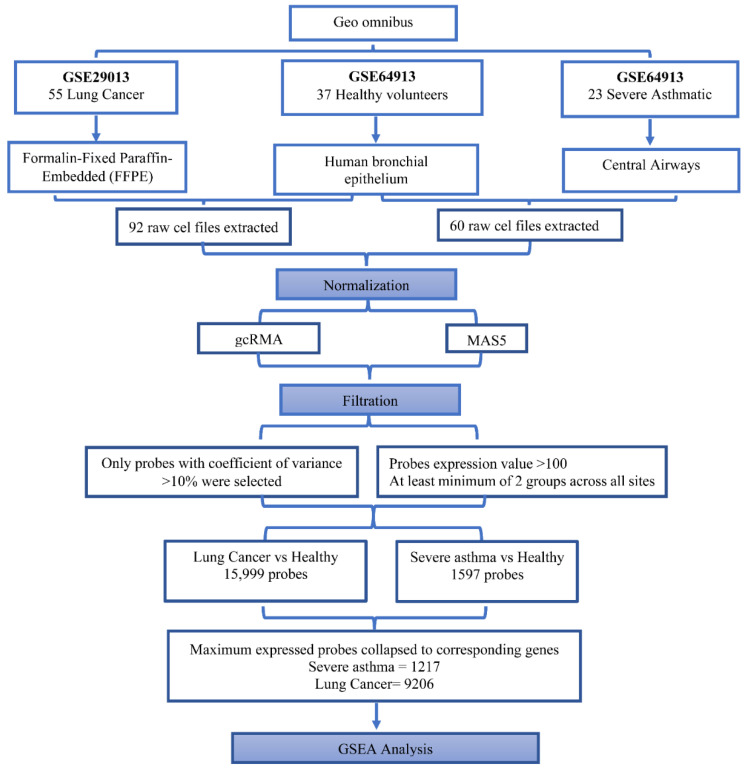
Flowchart outlining the steps of the bioinformatics approach used to identify differentially expressed genes in severe asthmatic bronchial epithelium compared to healthy controls and lung cancer compared to healthy controls. Abbreviations: GEO omnibus, Gene Expression Omnibus; gcRMA, guanine cytosine Robust Multi-Array Analysis; MAS5, Affymetrix Microarray Suite 5.

The Affymetrix Human Genome U133 Plus 2.0 Microarray chip has 54,675 probes, where each gene is represented by more than one probe. Raw Affymetrix CEL files (*n* = 60, 23 severe asthmatic, and 37 healthy) were extracted from the GSE64913 dataset and normalized using an in-house algorithm developed by Hamoudi et al. [16]. Briefly, the guanine cytosine Robust Multi-Array Analysis (gcRMA) and Affymetrix Microarray Suite 5 (MAS5) packages of the R Bioconductor statistical software version 3.6.3 were applied to normalize and remove the background noise. gcRMA and MAS5 expression values were used for the next nonspecific filtering based on the coefficient of variation (CV). The CV was calculated as the mean/standard deviation of each probe across all cases. Subsequently, nonspecific filtering was performed to remove nonvariant genes, and only the probes with MAS5 values ≥ 50 and CV values of 10–100% in the gcRMA across all cases were passed and intersected to obtain a common set of variant probes. The enriched genes identified from the differentially activated pathways were mapped to the raw data of the genes, and the fold change was calculated. The flowchart of the entire workflow is presented in Figure 2.

#### 2.2.2. Gene Set Enrichment Analysis for the Differentially Expressed Pathways among Severe Asthmatics and Healthy Controls

**Figure 3:**
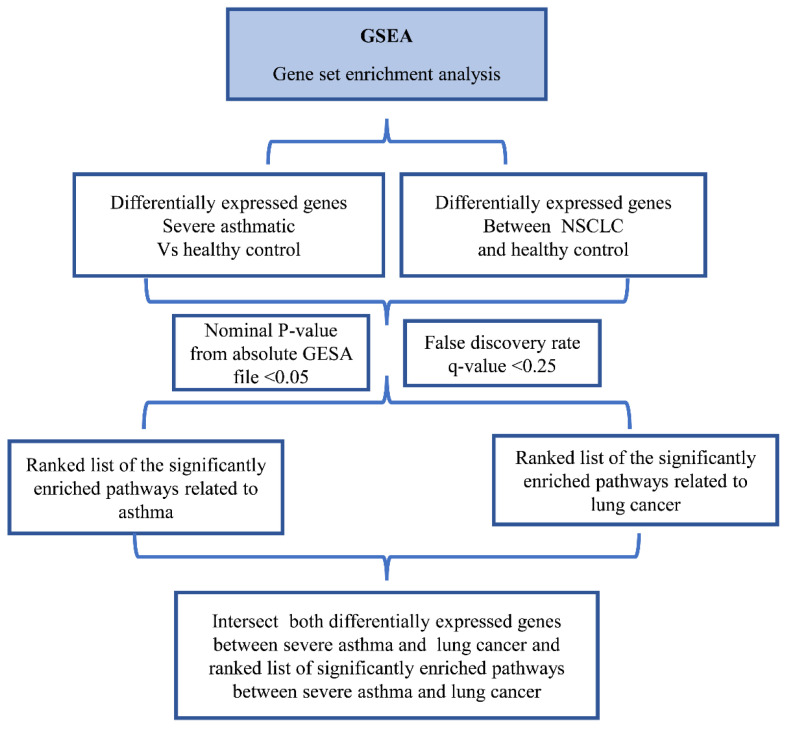
Flowchart of the bioinformatics approach used to identify gene sets related to severe asthma and lung cancer.

The filtered probes were annotated and collapsed to their corresponding genes using the GSEA software (http://software.broadinstitute.org/gsea/downloads.jsplast access date was 24 December 2021) by choosing probes with the maximum expression of each gene [29]. The control probes, along with those not assigned to a gene, were excluded. Hence, the resultant filtered probes were only the variant probes as per the GSEA manual. The filtered probes were collapsed to genes and used as input for the GSEA to identify the significantly enriched pathways among sets related to the C5 Gene Ontology (GO) gene set collection C5 biological process (BP) (c5. go.bp. v7.2. symbols) and C5 molecular function (MF) (c5.go.mf.v7.2. symbols). The results of the GSEA were ranked according to the nominal *p*-values, which were identified through absolute GSEA (<0.05), and the false discovery rate (≤0.25) as described previously [16,30] (Figure 3). In order to reduce the dataset, a systematic cross-reference of each gene enriched within statistically significant pathways was carried out. The genes with the highest frequency across the multiple significant pathways were compared across the disease phenotypes.

#### 2.2.3. Microarray Data Analysis to Identify Genes Differentially Expressed between NSCLC Patients and Healthy Controls

Two datasets were selected: GSE29013 for lung cancer patients and GSE64913 for healthy controls. Raw CEL files (*n* = 92) for 55 NSCLC and 37 healthy patients were extracted, and the processing was performed as detailed in Figure 2. The processed probes with >10% CV and >100-fold expression were filtered and further used for GSEA.

#### 2.2.4. Gene Set Enrichment Analysis for the Differentially Expressed Pathways among NSCLC Patients and Healthy Controls

The filtered probes for the lung cancer dataset compared to healthy controls were processed as detailed previously for severe asthmatics. The 15,999 probes filtered among the lung cancer dataset were collapsed to a list of 9206 genes, and the GSEA was performed as described earlier for severe asthmatics (Figure 3).

#### 2.2.5. In Silico Identification of Intracellular Pathways among Asthmatic and NSCLC Patients in Comparison to Healthy Controls

In order to identify the common pathways comprising most of the identified genes in the gene set analysis, Metascape (http://metascape.orglast access date 15 January 2022) was used to extract the top enriched pathways that were either upregulated or downregulated in severe asthma and/or lung cancer compared to the healthy controls.

### 2.3. Molecular Validation

#### 2.3.1. RNA Extraction

Total RNA was isolated from formalin-fixed paraffin-embedded (FFPE) blocks as previously described [31] from three groups—(1) severe asthma (AS), (2) lung cancer (LC), and (3) asthmatic cases who developed lung cancer (AC)—using the Recover All total nucleic acid isolation kit as per the manufacturer’s protocol (Invitrogen, Waltham, MA, USA), followed by DNA digestion using Turbo DNase (Invitrogen, Waltham, MA, USA ).

The total RNA extraction from the plasma and cell lines was performed using TRIzol reagent and the Pure link RNA extraction kit (Thermo Fisher Scientific, Waltham, MA, USA) according to the manufacturer’s instructions. RNA was quantified using the Nanodrop 2000 Spectrophotometer (Thermo Fisher Scientific, USA).

#### 2.3.2. cDNA Synthesis Using Gene-Specific Primer and Random Primer

The Superscript first-strand synthesis system for RT-PCR (Invitrogen, Cat. No. 11904-018) was used for cDNA synthesis for RNA obtained from FFPE samples. cDNA synthesis was carried out using gene-specific primers in three batches to include all the genes of interest. To prepare the RNA/primer mixes, for each run, ~1 μg of amplified RNA was taken from each sample, 1 μL of 1 pmol of nine different reverse primers per run (sequences in Supplementary Table S1) were mixed, and 1 μL of 10 mM dNTP was added; the volume was made up to 10 μL with nuclease-free water. The samples were then incubated in a thermocycler (Eppendorf) at 65 °C for 5 min and immediately placed on ice for at least 2 min. The reaction mixture for reverse transcription was prepared by mixing 2 μL of 10× RT buffer, 4 μL of 25 mM MgCl_2_, 2 μL of 0.1 M DTT, 1 μL of RnaseOUT enzyme, and 1 μL of Superscript III enzyme. The total 10 μL of the reaction mixture was added to the RNA/primer mix previously prepared. The samples were then incubated at 50 °C for 50 min, followed by reaction termination at 85 °C for 15 min.

For plasma and cell line RNA, cDNA was synthesized using the high-capacity cDNA synthesis kit (Applied Biosystems, Waltham, MA, USA) according to the manufacturer’s protocol. The kit contains RT random primer for cDNA preparation from both mRNA and rRNA

#### 2.3.3. Quantitative Reverse Transcription PCR (RTq-PCR)

The expression of the genes identified by in silico analysis was validated by RTq-PCR for cDNA obtained from the archival tissue biopsies, plasma, and cells. Approximately 50 ng of the gene-specific cDNA obtained from AS, LC, and AC tissues, as well as lung cancer and asthmatic cells and plasma, was added to 2× maxima SYBR green master mix (Thermo Fisher Scientific, Waltham, MA, USA) along with the primers as listed in Supplementary Table S1. The reaction was carried out in a Quant Studio 3 cycler (Thermo Fisher Scientific, Waltham, MA, USA) according to the manufacturer’s instructions. The cycling conditions were an initial single hold stage at 50 °C for 2 min, 95 °C for 10 min, and then 40 cycles of 95 °C for 15 s, 60 °C for 1 min, and 95 °C for 15 s; there was then a melt-curve stage: 60 °C for 1 min and 95 °C for 1 s. Each cDNA reaction was performed in triplicate, and each experiment was repeated three times along with a negative cDNA sample and a negative non-template control for each pair of primers. The Ct value of the gene of interest was normalized against the expression of the housekeeping gene (18S) for each sample, and the relative gene expression (2^−ΔΔCt^) was derived from the ΔCt values [32,33]. The fold-expression values were normalized to log_2_, and the relative expression of each gene was compared between the groups.

### 2.4. Statistical Analysis

Statistical analysis of the experimental data was performed from independent experiments using the SPSS software version 23, and the Mann–Whitney test was used to determine significance; *p* < 0.05 was considered statistically significant. The box plots for all the analyses were prepared using the GraphPad Prism software (version 8).

## 3. Results

### 3.1. In Silico Identification of Significant Gene Sets and DEGs between Severe Asthma and Lung Cancer Patients versus Healthy Controls

The transcriptomic datasets available in the public domain for severe asthma and healthy controls (GSE64913) and the lung cancer patients (GSE29013) were used to identify the differentially expressed genes (DEGs) among severe asthmatics and lung cancer patients.

Expression analysis of the microarray data for 23 severe asthmatic, 55 lung cancer, and 37 healthy controls was performed using nominal *p*-values from the absolute GSEA file <0.05 and a false discovery rate *q*-value < 0.25. From the analysis, 1597 probes were filtered from the 54,675 probes present in the Affymetrix Human Genome U133 Plus 2.0 Microarray chip. These 1597 probes were collapsed to 1217 genes for severe asthmatic patients versus healthy controls. For the lung cancer versus healthy control set, 15,999 probes were obtained after filtration and collapsed to 9206 genes, as shown in Figure 2.

**Figure 4:**
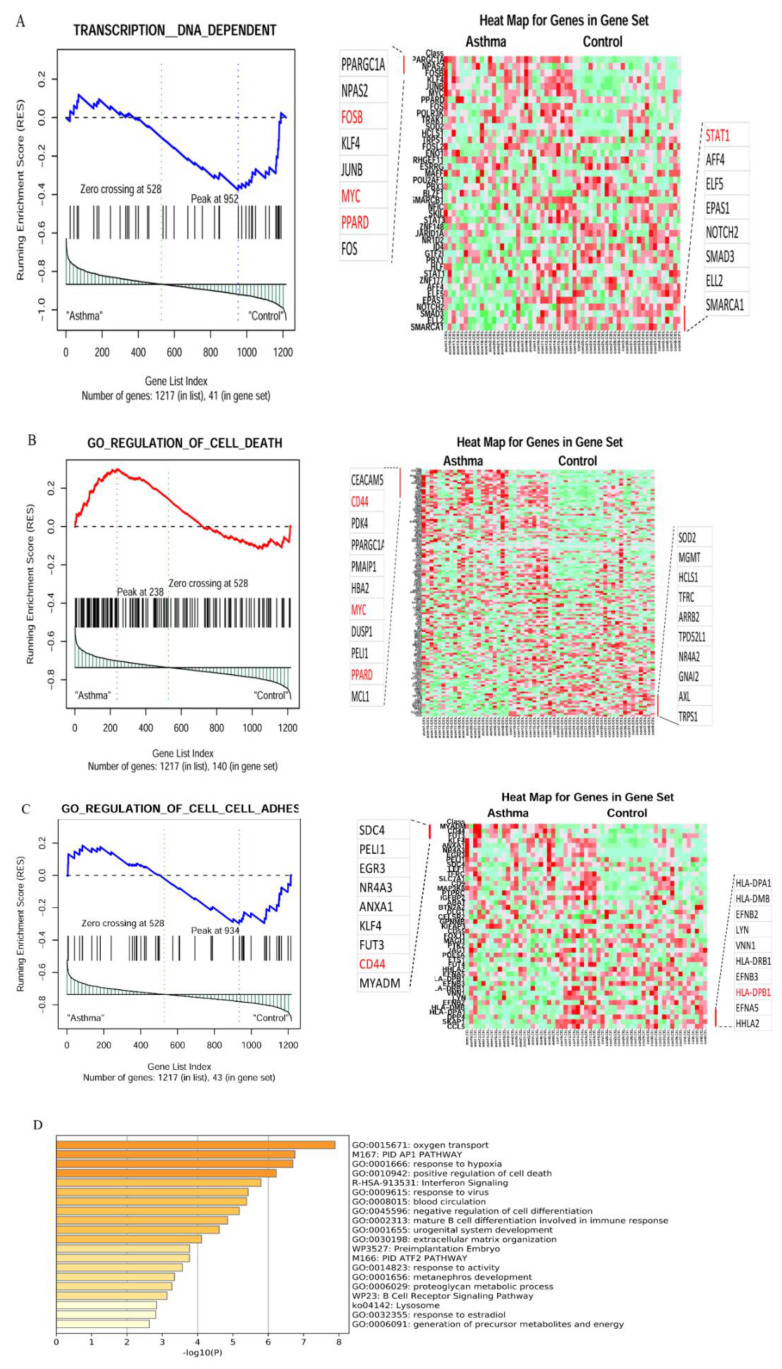
Gene set enrichment analysis (GSEA) of the differentially expressed genes between severe asthmatic bronchial epithelium (*n* = 23) and healthy bronchial epithelium (*n* = 37) in GSE64913. (**A**) DNA transcription, (**B**) regulation of cell death, (**C**) regulation of cell adhesion (left panel shows the distribution of DNA transcription, regulation of cell death, and cell adhesion target genes according to their rank position. The right panel shows a heatmap illustration of their expression between asthmatic and healthy control). (**D**) The top enriched pathways whether upregulated or downregulated in severe asthma compared to healthy controls using metascape (http://metascape.org last access date 15 January 2022): a gene annotation and analysis online resource that generates a graphical representation.

The results show that the most interesting gene sets were the annotated gene ontology sets C5 BP and C5 MF. Using the GSEA methodology from those two sets identified differentially activated cellular pathways between severe asthmatics and healthy controls. In total, 101 significantly enriched gene sets were identified (*p* = 0.05 and FDR = 0.25). These could be broadly classified into the following categories: signal transduction of apoptosis, regulation of cell adhesion, transcription and protein modification, metabolic processes or cell motility, and miscellaneous (Table 5). Analysis of the leading-edge genes underlying the enrichment of each individual gene set revealed that many were consistently represented, suggesting that they strongly influenced the expression pattern in severe asthmatics (Figure 4A–C).

The microarray data from 92 patients (55 lung cancer and 37 healthy controls) using the gene set GSE29013 and the absolute GSEA revealed the enrichment of transcripts among the main pathways that contribute to tissue and structure morphogenesis, regulation of cell death, transcription, and protein modification (Table 6).

In total, 628 genes differentially expressed between severe asthmatics and healthy controls (527 upregulated and 101 downregulated) were identified with a fold-change cutoff of 1.0 for upregulated and 0.75 for downregulated genes. The fold-change calculation was carried out by mapping the enriched genes to the raw Affymetrix gene expression values (Supplementary Data S2 and S3).

**Figure 5:**
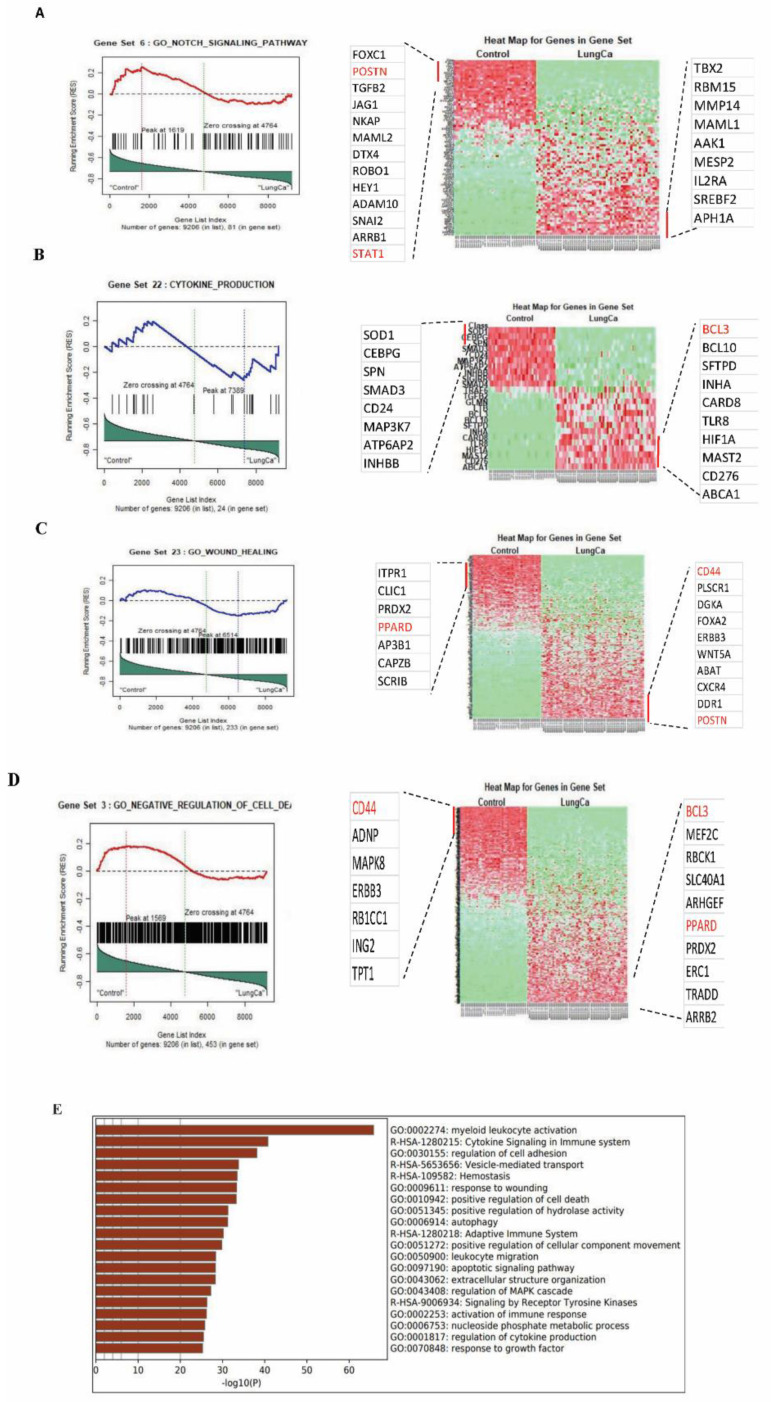
Gene set enrichment analysis (GSEA) of the differentially expressed genes between lung cancer bronchial epithelium (*n* = 55) in GSE29013 and healthy bronchial epithelium (*n* = 37) in GSE64913. (**A**) Notch signaling pathway, (**B**) cytokine production, (**C**) wound healing, (**D**) negative regulation of cell death (left panel shows the distribution of notch signaling pathway, cytokine production, wound healing, negative regulation of cell death according to their rank position. The right panel shows a heatmap illustration of their expression between lung cancer and healthy control). (**E**) The top enriched pathways, whether upregulated or downregulated in lung cancer, compared to healthy controls using metascape (http://metascape.org last access date 15 January 2022): a gene annotation and analysis online resource that generates a graphical representation3.2.

Overall, 6593 genes were identified that were differentially expressed among lung cancer patients in comparison to healthy controls with a fold-change cutoff of 1.5 for upregulated and 0.5 for downregulated genes (3635 upregulated and 2958 downregulated) (Supplementary Data S2 and S3). The leading-edge analysis provided details on the genes consistent across the gene sets, indicating their likely involvement in lung cancer pathogenesis (Figure 5A–D).

### 3.2. In Silico Validation of Differentially Activated Pathways Using Metascape Analysis

**Figure 6:**
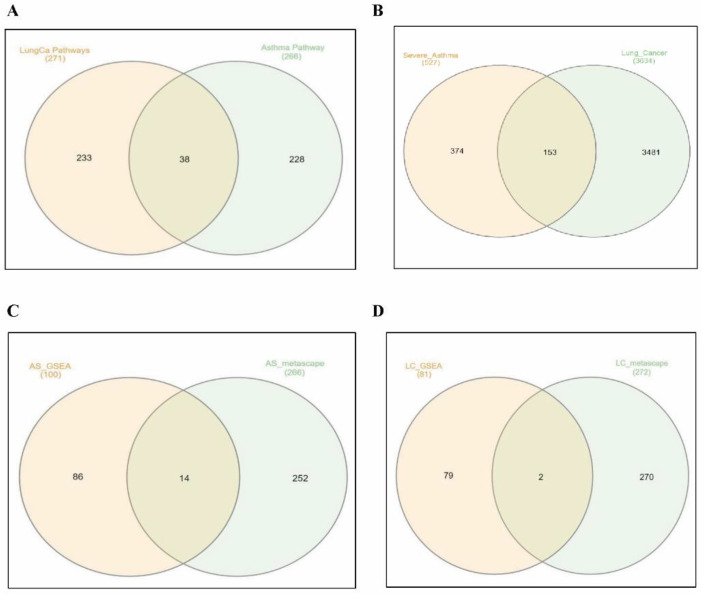
Venn diagram showing common pathways and genes among lung cancer patients and severe asthmatics. (**A**) Common pathways between asthmatics and lung cancer patients from Metascape analysis. (**B**) Commonly upregulated genes between asthmatics and lung cancer groups. Common pathways occurring between Metascape and GSEA analysis for (**C**) severe asthmatics and (**D**) lung cancer patients.

In order to cross-validate the results obtained from GSEA, Metascape analysis was carried out for the upregulated genes in the differential transcriptome of the asthmatic samples and lung cancer samples, revealing the top enriched pathways as implicated in Figure 4D and Figure 5E, respectively (Supplementary Data S4 and S5). The Venn output revealed that 38 pathways were commonly activated among both datasets, and 153 genes were commonly upregulated in both lung cancer patients and asthmatics (Figure 6A,B).

From the Metascape analysis and the top enriched transcripts obtained, the genes overrepresented among both asthmatics and lung cancer patients were determined (Table 7 and Table 8). The DEGs identified in common across the datasets are highlighted in bold.

Among the asthmatic clusters, pathways related to cell adhesion, extracellular matrix organization, and interferon signaling were enriched (Figure 4D and Table 5). These regulatory pathways are, in general, associated with the inflammatory and tissue remodeling events involved in asthma pathobiology.

A comparative set theory performed on the pathways identified by both GSEA and Metascape analysis revealed commonly identified key pathways among severe asthmatics (Figure 6C). These important pathways include those related to cell adhesion and epithelial cell proliferation, which are known to be modulated during tissue remodeling in asthmatics (Supplementary Table S2). Hence, the data obtained from both methods were validated.

Similarly, the lung cancer dataset analysis showed the activation of key pathways such as receptor tyrosine kinase signaling, growth factor response, blood-vessel development, and cell adhesion (Figure 5E and Table 6).

### 3.3. Gene Expression Analysis from the Microarray Datasets for Severe Asthmatics and Lung Cancer Patients

**Figure 7:**
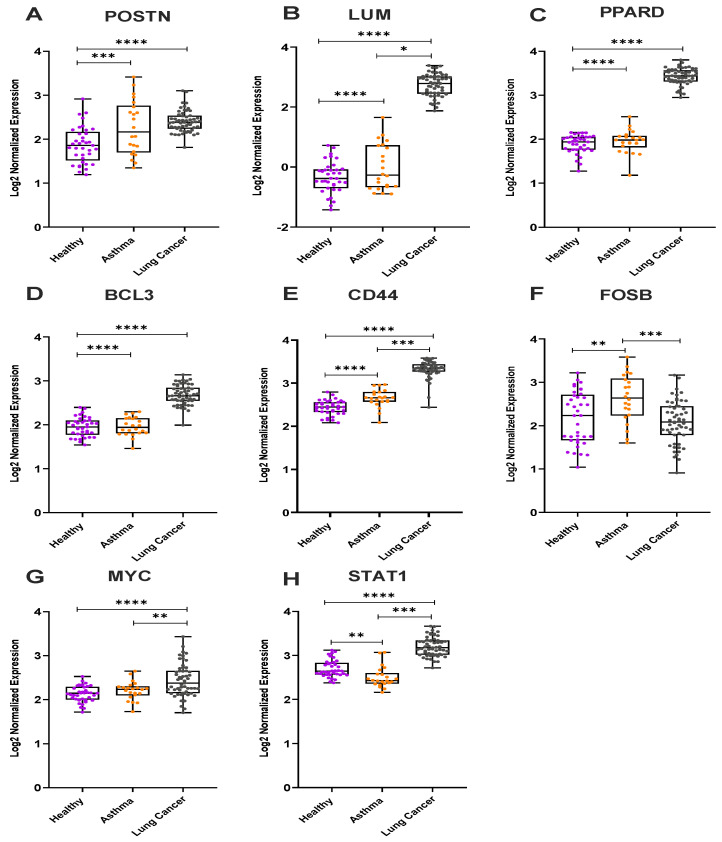
Boxplots for the differentially expressed genes in severe asthma and lung cancer from Microarray dataset. (**A**) *POSTN* (**B**) *LUM* (**C**) *PPARD* (**D**) *BCL3* (**E**) *CD44* (**F**) *FOSB* (**G**) *MYC* (**H**) *STAT1* (Mann −Whitney test, * *p* < 0.05, ** *p* < 0.01, *** *p* < 0.001, **** *p* < 0.0001).

The results from the GSEA and metascape analysis of the microarray data led to the identification of eight genes (by fold change and frequency count methods) overrepresented in severe asthmatics and lung cancer patients. Two genes, periostin (*POSTN*) and lumican (*LUM*) were upregulated in both severe asthmatics and lung cancer patients respectively (Figure 7A,B and Supplementary Table S3). The other six genes, peroxisome proliferator-activated receptor delta (*PPARD*), B-cell lymphoma 3 (*BCL3*), cluster of differentiation 44 (*CD44*), protein fosB (*FOSB*), myelocytomatosis (*MYC*), and signal transducer and activator of transcription 1 (*STAT1*), were detected with high frequency among severe asthmatics and lung cancer patients (Supplementary Table S4) in the leading-edge analysis. These genes were observed to be differentially expressed among the asthmatics and lung cancer patients in comparison to healthy controls, as presented in Figure 7. The fold changes for each gene among both datasets are listed in Supplementary Table S3.

The other genes, *CD44*, *PPARD*, and *STAT1*, were also observed to be highly upregulated in lung cancer samples compared to asthmatics and healthy controls (Figure 7A–H). In addition to *POSTN* (a known prognostic marker for asthma), *FOSB* can be observed to be upregulated in asthmatics (Figure 7G).

As CD44 is known to occur in different isoforms, analysis for a specific probe for each variant was performed. Supplementary Data S6 lists the probe ID and descriptions for targets. However, from the datasets studied here, the expression for only four probes could be retrieved. Supplementary Figure S1 shows that all four probes show an increase in expression in Lung cancer samples.

### 3.4. In Vivo Validation Using Archival Biopsies by RT-qPCR

**Figure 8:**
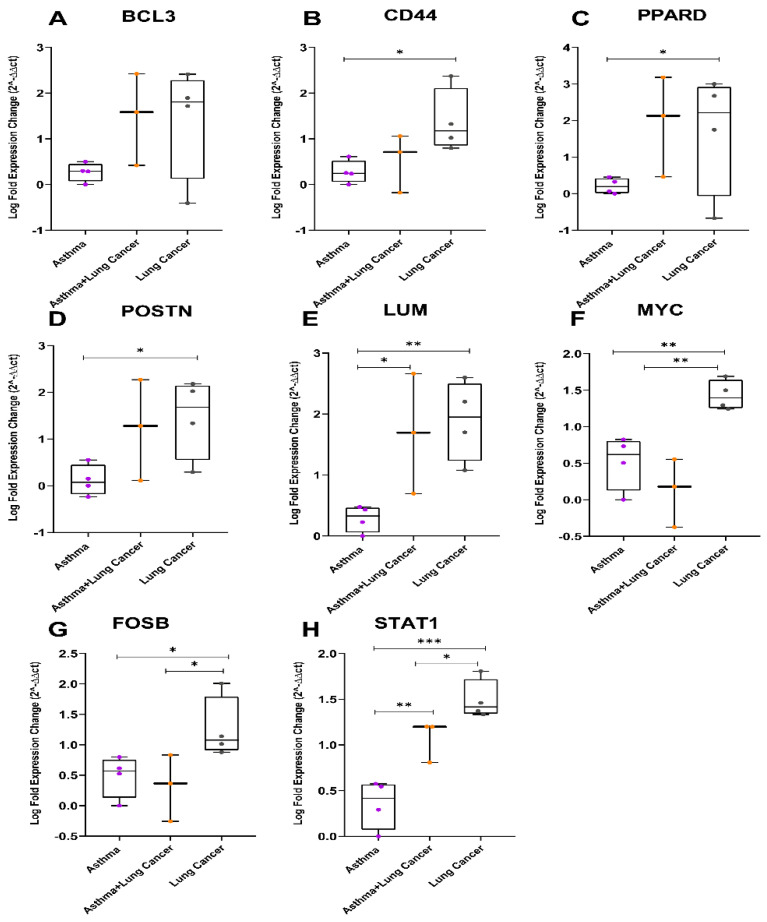
Boxplots for the differentially expressed genes among severe asthmatics, lung cancer and asthmatics with lung cancer development validated by RT-qPCR analysis in archival tissue biopsies. (**A**) *BCL3*, (**B**) *CD44*, (**C**) *PPARD*, (**D**) *POSTN*, (**E**) *LUM*, (**F**) *MYC*, (**G**) *FOSB*, and (**H**) *STAT1* (Mann −Whitney test, significance * *p* < 0.05, ** *p* < 0.01, *** *p* < 0.001).

To validate the findings of the microarray analysis, the six genes identified (*PPARD*, *BCL3*, *CD44*, *FOSB*, *MYC*, and *STAT1*), along with *POSTN* and *LUM*, were assessed using RT-qPCR in 11 archival tissue biopsies from four severe asthmatics (AS), three asthmatics who developed lung cancer (AC), and four lung cancer (LC) patients. The RT-qPCR data revealed a significant increase in gene expression levels for *BCL3*, *LUM*, *PPARD*, *POSTN*, and *STAT1* among the LC group (Supplementary Table S5) and a trend toward an increase in AC compared to the AS group. *CD44*, *FOSB*, and *MYC* were highly upregulated in LC compared to the AS and AC groups, as predicted (Supplementary Table S5). The primers used to analyze CD44 expression were designed in a common exon region shared by all the isoforms or transcript variants. The data suggests that the DEGs identified by the microarray study were consistent with the qPCR analysis for tissue biopsies (Figure 8A–H).

### 3.5. Relative Gene Expression of the Eight Genes in Plasma Samples

**Figure 9:**
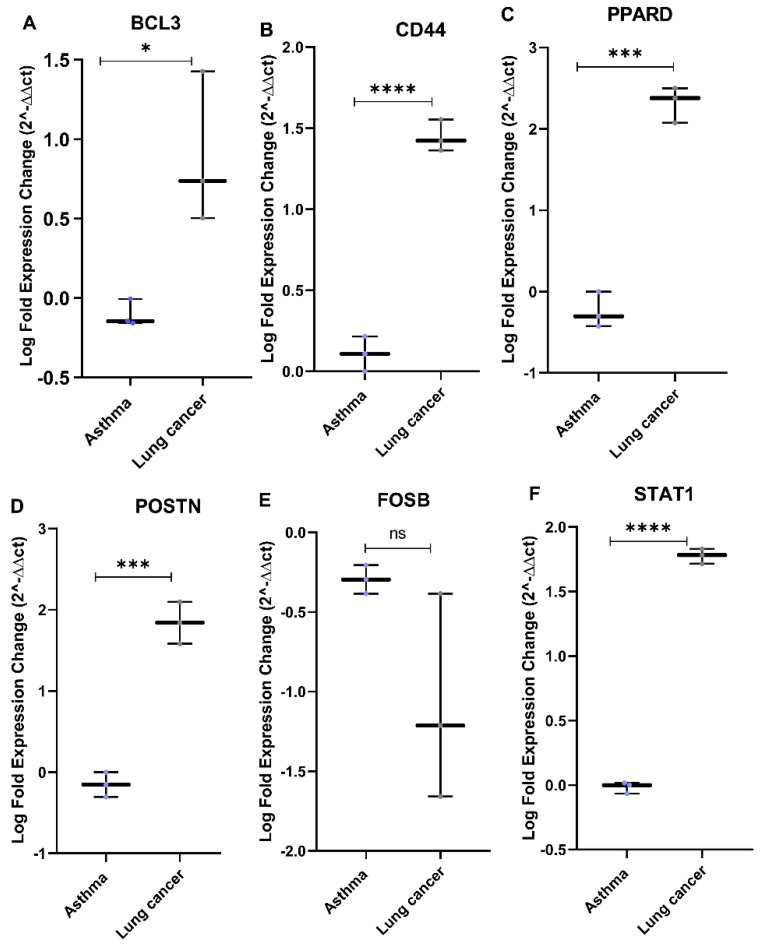
Boxplots for the differential expression of genes identified in silico in plasma samples collected from asthmatics and lung cancer patients. (**A**) *BCL3*, (**B**) *CD44*, (**C**) *PPARD*, (**D**) *POSTN*, (**E**) *FOSB*, and (**F**) *STAT1* (Mann −Whitney test, significance * *p* < 0.05, *** *p* < 0.001, **** *p* < 0.0001, ns—not significant).

The relative gene expression for the plasma samples collected from severe asthmatics and lung cancer patients was tested for the eight genes predicted in silico. The fold change in expression showed significant upregulation for the genes *BCL3*, *CD44*, *PPARD*, *POSTN*, and *STAT1* in lung cancer patients compared to asthmatics (Figure 9A–C,F). In the case of *FOSB* (Figure 9E), the plasma showed a variation from the tissue biopsy, whereby the relative expression of this gene was higher in lung cancer patients than in asthmatics (Supplementary Table S6).

### 3.6. In Vivo Validation Using Independent NSCLC Patient Cohort

**Figure 10:**
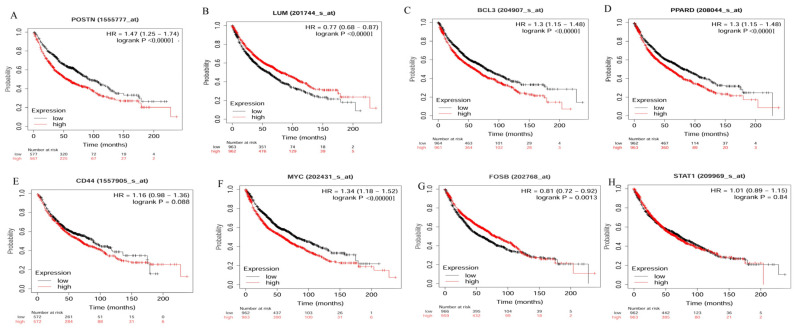
In vivo validation for the effect of eight genes on overall survival in lung cancer patients using KM Plot. (**A**) *POSTN*, (**B**) *LUM*, (**C**) *BCL3*, (**D**) *PPARD*, (**E**) *CD44*, (**F**) *MYC*, (**G**) *FOSB*, and (**H**) *STAT1*. HR = Hazard ratio.

The survival pattern for the genes *POSTN*, *LUM*, *BCL3*, *PPARD*, *CD44*, *MYC*, *FOSB*, and *STAT1* in an independent NSCLC patient cohort of 1925 samples was analyzed using the KM plot [19], as described in Section 2. The survival curve shown in Figure 10 reveals that a higher expression of the genes *POSTN*, *PPARD*, *BCL3*, and *MYC* denotes poor survival among lung cancer patients. In contrast, for the genes *LUM* and *FOSB*, lower expression in lung cancer patients denote poor survival (Figure 10). *CD44* and *STAT1* showed a nonsignificant log p-rank value (Supplementary Table S7). However, analysis for probes specific for variants for CD44 showed no variation in survival plots where increase in expression did not benefit overall survival (Supplementary Figure S2). 

In addition, the effect of sex, smoking, and cancer stage on the survival pattern for each gene was performed. Interestingly, a significant effect on the gene expression and survival pattern was observed in the case of *BCL3*, *PPARD*, and *MYC*, where poorer survival was observed among males and smokers either in stage 1 or 2 (Supplementary Table S9 and Supplementary Figure S3). On the other hand, decreased mortality was observed with an increase in the expression for the genes *POSTN*, *FOSB*, and *LUM,* mainly in males diagnosed with early-stage lung cancer either 1 or 2 (Supplementary Table S9 and Supplementary Figure S4). 

### 3.7. In Vitro Validation Using Asthmatic and Lung Cancer Cell Lines

**Figure 11:**
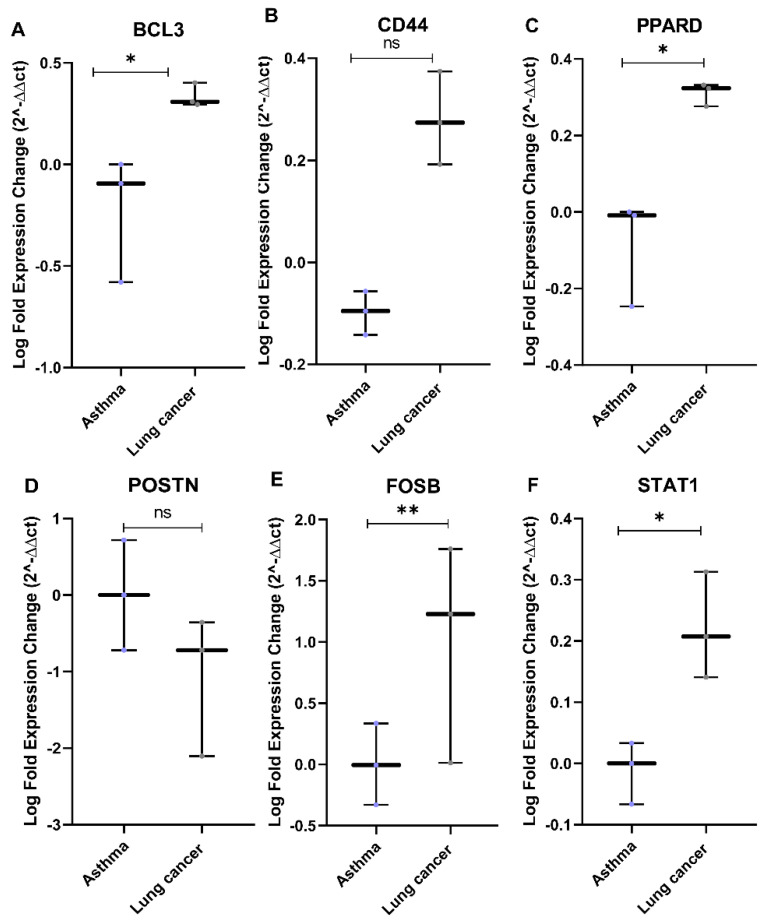
Boxplots for the differential expression of the genes identified in silico in different asthmatic and lung cancer cell lines. (**A**) *BCL3*, (**B**) *CD44*, (**C**) *PPARD*, (**D**) *POSTN*, (**E**) *FOSB*, *and* (**F**) *STAT1* (Mann −Whitney test, significance * *p* < 0.05, ** *p* < 0.01, ns—not significant).

The differential expression pattern for the eight genes identified in silico was examined in asthmatic and lung cancer cell lines (Supplementary Table S8). The fold changes in gene expression in lung cancer relative to asthmatic cell lines were in line with the in silico prediction for the genes *BCL3*, *CD44*, *PPARD*, *POSTN*, *FOSB*, and *STAT1* (Figure 11). All the genes except *POSTN* showed higher expression in lung cancer cells (A549, SK-Lu-1, and Calu3). Asthmatic cells (DHBE, S13, and S14) displayed higher *POSTN* expression, as expected (Figure 11D).

## 4. Discussion

The present study aimed to identify genes that putatively indicate the early transition of the severe inflammatory state observed in severe asthmatic cases, which may also be involved in the early progression of lung cancer.

In silico analysis using the gene set enrichment analysis (GSEA) of asthmatic and lung cancer microarray datasets revealed essential pathways in their respective pathophysiology. Interestingly, both datasets showed enrichment of transcripts that contribute to tissue and structure morphogenesis.

One of the common pathways in asthma is the humoral immune response, a fact supported by different studies related to the role of Th2 immunity in the immunopathology of asthma, which influences the severity of the condition [34]. In addition, the results identified pathways associated with asthma pathophysiology, including interferon signaling, which is involved in the antiviral host response, the type 2 immune response for environmental triggers such as allergens, and stress, which in turn disrupts the bronchial epithelium, which activates the wound-healing response [35,36].

In the lung cancer dataset, pathways related to the response to stress and DNA repair were enriched, which is supported by studies indicating the association of a somatic and germline mutation in a DNA-repair gene with lung adenocarcinoma among 2.5% of the cancer cases tested [37,38,39]. The intersection of the pathways using both GSEA and hypergeometric analysis (implemented using Metascape) identified 14 important pathways for the severe asthma group, including cell adhesion and epithelial cell proliferation. A similar comparison identified the wound-healing pathway in lung cancer.

The common key pathways upregulated in both functional clusters for asthma and lung cancer datasets were events related to cell adhesion, extracellular matrix organization, and growth factor response. These mechanisms are important in the pathobiology of both diseases. Hence, the genes functioning among these clusters overrepresented in both datasets were selected to determine molecular markers at the intersection of the transition from severe asthma to lung cancer.

The expression of eight genes we retrieved from both datasets (*BCL3*, *CD44*, *FOSB*, *LUM*, *MYC*, *PPARD*, *POSTN*, and *STAT1*) was confirmed by the differential expression in FFPE biopsies from patients. Interestingly, the histopathology of asthma, lung cancer, and asthma with lung cancer suggests that asthma and lung cancer exhibit similar molecular mechanisms and pathways for the progression and/or increased risk of lung cancer in severe asthma. This association was recently shown to be the case using epidemiological data linking the two diseases [14,40].

We validated in silico data at multiple levels using FFPE, plasma, and cell lines from both severe asthmatics and lung cancer patients. The gene expression pattern of the eight genes in the tissue biopsies clearly ascertains the role of these genes in a specific disease state as implicated in the functional pathway analysis. The severe inflammatory state among the severe asthmatics can initiate the pathobiological events that result in lung cancer [41]. The results obtained from this study showed that the genes differentially expressed in both the lung cancer and the asthma datasets from in silico analysis were differentially expressed at the tissue and cellular levels, as indicated by the validation results using cell lines and plasma samples, which echoed the in silico observations.

The differential expression pattern seems to be implicated in both asthma and lung cancer, suggesting a possible common molecular mechanism between the two diseases. In particular, the genes *BCL3*, *CD44*, *PPARD*, and *STAT1* showed an increase in expression among the mixed group (asthmatics diagnosed with lung cancer) and lung cancer samples.

BCL3, an IkB member, interferes with the heterodimerization of NF-κB subunits, thereby inhibiting the transcription of proinflammatory genes. On the other hand, higher mRNA and protein expression of BCL3 is associated with overall survival among NSCLC patients in stages 1 and 2 [42].

STAT1 is a known transcription factor with roles in both asthma and lung cancer pathogenesis. STAT1 is activated by IL-4, IL-13, and IFNγ and forms either a homodimer or a heterodimer with other STAT proteins to induce the expression of genes related to apoptosis and immune-suppressive cytokines [43,44,45]. Among the STATs, STAT1 mRNA expression was observed to be high in NSCLC compared to normal tissue [46], and it is known to exhibit antitumor activity. The dual nature of STAT1 as a tumor suppressor and tumor promoter has been addressed in many studies, but no clear mechanistic details have been provided to elucidate the duality [47]. A few reports have postulated that the isoforms of STAT1 could be differentially activated owing to either suppression or tumor progression [48,49]. Some studies have mentioned that the expression levels of STAT1 and pSTAT1 in specific cell types could be prognostic markers for cancer progression [50,51].

PPARD is also involved in inhibiting the transcription of NF-κB target genes by disrupting the heterodimer formation of NF-κB subunits. The protumorigenic capabilities of PPARD were clearly described by Wagner and Wagner (2020). As BCL3 and PPARD are known to be associated with NF-κB [52,53] and have functional roles in cell adhesion, inflammation, proliferation, and cancer progression, they could be key modulators in the pathological changes at sites of tissue injury. A study showed that STAT1 interacts with PPARγ in the induction of CD36 expression. STAT1 acetylation, which is controlled by p300, is required for STAT1’s interaction with PPARγ [54].

*POSTN* (a known biomarker for asthma) was upregulated in the mixed group compared to asthma and lung cancer samples. *LUM* was upregulated in the lung cancer dataset alone, as seen in the case of microarray analysis. Hence, it was not considered as a differential marker between lung cancer and asthma. Notably, *POSTN*, an important biomarker for Th2-type asthma and a negative prognostic biomarker for lung cancer [55,56,57], was observed to be upregulated in the mixed group. Periostin is an extracellular matrix protein and is known to be involved in the epithelial–mesenchymal transition, a key mechanism in the initial stages of cancer pathogenesis [56]. Moreover, the expression levels decreased among the lung cancer samples compared to the mixed group, indicating its probable involvement in the early development of lung cancer from an asthmatic state. Similarly, *BCL3*, *CD44*, *PPARD*, and *STAT1* were upregulated in both the mixed group and lung cancer samples; thus, they may also be involved in the transition. Lumican, also an extracellular matrix protein, is involved in cell adhesion and migration, similar to periostin [58].

The survival analysis for the eight genes was assessed in KM Plotter among the lung cancer samples, which re-emphasized the fact that a higher expression of *POSTN*, *BCL3*, *PPARD*, and *MYC* could be considered a marker for poor survival among lung cancer samples. No correlation for the survival rate was observed in the case of *STAT1*, as demonstrated in other studies [46].

Taken together, the molecular pathways and genes identified in this study are known to be involved in various pathobiological events in severe asthma and lung cancer. Collectively, the literature reveals that overlapping events in both disease conditions are due to the common site of injury: the airway and bronchial epithelium. Although the starting point of lung cancer may not be the asthmatic injury of the lungs, the incidence of lung cancer among severe asthmatics is due to the state of chronic inflammation of the lung [59]. However, if the molecular markers identified in the study are indicative of a transition from a severe inflammatory state of the lung affected with asthma toward an early lung cancer condition, they could be used to screen patients for early stages of lung cancer.

In summary, the key molecular targets identified from this study can be potential predictors of early stages of lung cancer, as their evident role in severe asthma suggests an inflammation-induced cancer progression. The study used publicly available data to identify putative biomarkers, which were then validated using asthma and lung cancer tissue. Similarly, the validation in asthmatic and lung cancer cell lines reflected the observations from our in silico predictions. The differential expression patterns for the identified genes in plasma samples obtained from severe asthma and lung cancer patients further validate the findings. However, the results from this study warrant further investigation into the molecular mechanisms of the four genes (*PPARD*, *STAT1*, *BCL3*, and *POSTN*) in both asthma and lung cancer cell lines, independently and in combination. The results from these investigations may reproduce the findings from the present study and help to identify diagnostic and therapeutic targets for the early stages of lung cancer.

### Study Limitations and Justification

The main limitation of this study was the small sample size; however, this was circumvented by the large number of asthma and lung cancer samples in the in silico analysis that was used for the discovery of key targets linked to both asthma and lung cancer. Validation of the findings was conducted at multiple levels in plasma samples, in tissue biopsies, and in vitro using asthma and lung cancer cell lines. Considering that both in vitro and in vivo validation supported the findings from the in silico data, the genes identified may act as putative biomarkers for early lung cancer. However, this was a proof-of-concept study, and the findings require validation on a larger cohort to ascertain the differential expression of the seven transcripts. In addition, the findings from this study warrant further functional studies to characterize the role of the genes identified in the pathogenesis of asthma and lung cancer.

## 5. Conclusions

This study identified genes and pathways distinctly regulated in severe asthma and lung cancer using gene set enrichment analysis. The different etiologies of cancer as a genetic disease, and asthma caused by environmental factors, are reflected in their distinct pathways. In line with the hallmarks of cancer, receptor tyrosine kinase signaling wound healing and growth factors are activated in lung cancer and may be responsible for an increased risk of lung cancer in severe asthma. This study also identified unique pathways related to asthma, including adhesion, extracellular matrix, and epithelial cell proliferation. Analysis of the enriched genes derived from the pathway analysis identified seven genes present in both asthma and lung cancer: *BCL3*, *POSTN*, *PPARD*, *STAT1*, *MYC*, *CD44*, and *FOSB*. The validation of the genes using archival patient tissue biopsies, cell lines, and liquid biopsy samples revealed significant differential expression between asthma and lung cancer patients, providing possible insights into some of the molecular mechanisms involved in the pathogenesis between asthma and lung cancer. Subsequently, these transcripts may be potentially used as markers for early lung cancer and could be useful in preventing the progression to later stages of lung cancer.

## Figures and Tables

**Table 1:** Details on the samples and the subjects retrieved from the database.

Accession Number	GSE64913		GSE29013
	Severe Asthmatic (*n* = 17)	Healthy Control (*n* = 23)	Lung Cancer (*n* = 55)
Male	9	14	38
Female	8	9	17
No. of smokers	3	None	2
Age in years, mean (range)	41 (20–63)	26 (19–54)	63.5
Exacerbations	At least 2 per year	NA	NA
NSCLC stage	NA	NA	Stage 1 = 24 Stage 2 = 14 Stage 3 = 17

**Table 2:** Clinical characteristics of patients whose tissues were collected for FFPE blocks.

Clinical Variables	Disease		
	Severe Asthmatic (*n* = 4)	Asthmatic Patients That Developed Lung Cancer (*n* = 3)	Lung Cancer (*n* = 4)
Age in years; mean (range)	49 (32–61)	62 (26–83)	58 (55–91)
No. of males; *n* (%)	1 (25)	2 (66.6)	2 (50)
% FEV1; mean (range)	50.7 (38–61)	53 (43–64)	NA
Reversibility (% FEV1); mean (range)	16 (12–20)	21 (18–25)	NA
NSCLC Stage			
1 (%)	NA	1 (33.3)	
2 (%)	NA	1 (33.3)	1 (25)
3 (%)	NA	1 (33.3)	1 (25)
4 (%)	NA		2 (50)

**Table 3:** Characteristics of the patients from whom blood samples were collected.

Patient ID	Disease	Gender	Age	FEV1 (/L)
AS6	Asthma	Male	56	1.84
AS14	Asthma	Female	57	1.33
AS17	Asthma	Female	44	2.5
LC1	Lung cancer, stage 3	Male	58	-
LC2	Lung cancer, stage 4	Male	63	-
LC3	Lung cancer, stage 3	Male	77	-

**Table 4:** List of cell lines used in the study for molecular validation.

Cell ID	Description	Disease	Patient Details Gender, Age, Ethnicity	Catalog Number
A549	Lung epithelial	Lung cancer	Male, 58, Caucasian	C0016002
SK-LU-1	Lung epithelial	Lung cancer	Female, 46, Caucasian	C0016049
Calu3	Lung epithelial from metastatic site: pleura	Lung cancer; grade III epidermoid	Male, 25, Caucasian	C0016001
DHBE	Asthmatic epithelial cells	Asthma	Female, 54, Hispanic	00194911
S13	Epithelial cells retrieved from severe asthma patient	Severe asthma	Male, 53, East Asian	Isolated from the bronchial biopsy *
S14	Epithelial cells retrieved from severe asthma patient	Severe asthma	Female, 46, East Asian	Isolated from the bronchial biopsy *

* These cells were isolated from the bronchial biopsies collected from severe asthma patients at Rashid Hospital, Dubai. The FFPE blocks from the same tissues are mentioned above in Table 2.

**Table 5:** Gene sets differentially overrepresented in severe asthmatics vs. healthy controls.

Gene Sets	Size	Source	ES	NES	NOM *p*-Value	FDR *q*-Value	FWER *p*-Value	Tag %	Gene %	Signal	FDR (Median)	Glob. *p*-Value
Signal transduction												
CELL_CELL_SIGNALING	22	GO:0007267	0.4642	1.5824	0.0281	0.2633	0.3560	0.636	0.3890	0.3960	0.0000	0.0850
GO_RAS_PROTEIN_SIGNAL_TRANSDUCTION	17	GO_RAS_PROTEIN_SIGNAL_TRANSDUCTION	0.5485	1.6490	0.0123	0.0847	0.0670	0.529	0.3210	0.3640	0.0000	0.0580
GO_GTPASE_REGULATOR_ACTIVITY	20	GO_GTPASE_REGULATOR_ACTIVITY	0.4371	1.5238	0.0171	0.2652	0.5940	0.65	0.4600	0.3570	0.1522	0.0530
POSITIVE_REGULATION_ OF_CELL_ DEATH	22	GO Biological Processes	0.8167	1.7937	0.0000	0.0162	0.0239	0.682	0.1920	0.5610	0.0000	0.0060
GO_NEGATIVE_REGULATION_OF_CELL_ DEATH	90	GO_NEGATIVE_REGULATION_OF_CELL_DEATH	0.3999	1.5361	0.0381	0.3468	0.8680	0.533	0.4350	0.3260	0.2031	0.0730
Regulation of cell-to-cell adhesion												
GO_REGULATION_OF_CELL_CELL_ADHESION	43	GO_REGULATION_OF_CELL_CELL_ADHESION	0.4729	1.5837	0.0236	0.1378	0.1082	0.535	0.3820	0.3430	0.0000	0.0860
GO_POSITIVE_REGULATION_OF_CELL_ADHESION	41	GO_POSITIVE_REGULATION_OF_CELL_ADHESION	0.5554	1.9064	0.0000	0.2381	0.1430	0.439	0.2050	0.3610	0.0000	0.0800
GO_REGULATION_OF_CELL_SUBSTRATE_ADHESION	26	GO_REGULATION_OF_CELL_SUBSTRATE_ADHESION	0.5074	1.7722	0.0021	0.2739	0.4180	0.308	0.1310	0.2730	0.0000	0.0700
GO_BIOLOGICAL_ADHESION	134	GO_BIOLOGICAL_ADHESION	0.3695	1.6123	0.0022	0.3383	0.7640	0.44	0.3840	0.3050	0.1880	0.0710
GO_CELL_CELL_ADHESION	77	GO_CELL_CELL_ADHESION	0.4418	1.7117	0.0067	0.3622	0.5740	0.506	0.3820	0.3340	0.1444	0.0840
Transcription and protein modification												
TRANSCRIPTION	46	GO:0006350	0.4410	1.5196	0.0365	0.1963	0.4560	0.5	0.3590	0.3330	0.0000	0.0340
TRANSCRIPTION__DNA_DEPENDENT	41	GO:0006351	0.4573	1.5451	0.0340	0.1761	0.4210	0.537	0.3590	0.3560	0.0000	0.0270
GO_RNA_SPLICING	21	GO_RNA_SPLICING	0.4809	1.5485	0.0478	0.3423	0.8530	0.476	0.2770	0.3500	0.2007	0.0730
Miscellaneous												
GO_HUMORAL_IMMUNE_RESPONSE	16	GO_HUMORAL_IMMUNE_RESPONSE	0.5810	1.5893	0.0366	0.3531	0.7910	0.5	0.3340	0.3370	0.1959	0.0790
GO_HORMONE_TRANSPORT	23	GO_HORMONE_TRANSPORT	0.4065	1.4568	0.0387	0.3800	0.9210	0.304	0.1950	0.2500	0.2441	0.0790
GO_GLYCOSAMINOGLYCAN_BINDING	18	GO_GLYCOSAMINOGLYCAN_BINDING	0.6212	1.8600	0.0000	0.1535	0.0500	0.333	0.0970	0.3060	0.0000	0.0500

Abbreviations: ES, enrichment score; NES, normalized ES; NOM, nominal; FDR, false-discovery rate; FWER, family-wise error rate; Tag %, the percentage of gene tags before (for positive ES) of after (for negative ES) the peak in the running enrichment score; gene %, the percentage of genes in the gene list before (for positive ES) of after (for negative ES) the peak in the running enrichment score; GO, gene ontology.

**Table 6:** Gene sets differentially overrepresented in lung cancer patients vs. healthy controls.

Gene Sets	Size	Source	ES	NES	NOM *p*-Value	FDR *q*-Value	FWER *p*-Value	Tag %	Gene %	Signal	FDR (Median)	Glob. *p*-Value
Signal transduction												
GO_NOTCH_SIGNALING_PATHWAY	81	GO_NOTCH_SIGNALING_PATHWAY	0.3330	1.5135	0.0289	0.8450	0.2560	0.3210	0.2590	0.2400	0.0000	0.2540
REGULATION_OF_GENE_EXPRESSION	351	GO:0010468	0.2664	1.4706	0.0111	0.2621	0.8460	0.5160	0.5030	0.2670	0.1746	0.0270
SECRETORY_PATHWAY	48	GO:0045045	0.4318	1.6364	0.0163	0.3425	0.5770	0.3960	0.2740	0.2890	0.1337	0.1000
NEGATIVE_REGULATION_OF_APOPTOSIS	89	GO:0043066	0.3283	1.5110	0.0323	0.2598	0.7990	0.2920	0.2340	0.2260	0.1580	0.0310
NEGATIVE_REGULATION_OF_PROGRAMMED_CELL_DEATH	90	GO:0043069	0.3255	1.5061	0.0340	0.2575	0.8020	0.2220	0.1370	0.1940	0.1548	0.0290
Tissue and structure morphogenesis												
STRUCTURAL_CONSTITUENT_OF_RIBOSOME	31	GO:0003735	0.5982	1.7802	0.0119	0.4095	0.2390	0.5810	0.1530	0.4940	0.0000	0.1600
ORGAN_MORPHOGENESIS	54	GO:0009887	0.3809	1.4301	0.0383	0.2742	0.8840	0.4630	0.3450	0.3050	0.2011	0.0210
ORGAN_DEVELOPMENT	224	GO:0048513	0.3165	1.3987	0.0383	0.2833	0.9160	0.4380	0.3870	0.2750	0.2173	0.0220
Transcription and protein modification												
PROTEIN_CATABOLIC_PROCESS	35	GO:0030163	0.4445	1.8389	0.0021	0.4918	0.2090	0.5140	0.3060	0.3580	0.0000	0.1470
CELLULAR_PROTEIN_CATABOLIC_PROCESS	32	GO:0044257	0.4190	1.7027	0.0040	0.8381	0.4340	0.5000	0.3060	0.3480	0.0000	0.2620
PROTEIN_RNA_COMPLEX_ASSEMBLY	35	GO:0022618	0.4196	1.6975	0.0085	0.7046	0.4440	0.5710	0.3360	0.3810	0.0000	0.2330
Miscellaneous												
RESPONSE_TO_STRESS	252	GO:0006950	0.2780	1.4447	0.0439	0.2658	0.8740	0.4290	0.4290	0.2520	0.1908	0.0210
DNA_REPAIR	70	GO:0006281	0.3601	1.5256	0.0493	0.2525	0.7750	0.4430	0.3640	0.2840	0.1428	0.0320
CYTOKINE_PRODUCTION	24	GO:0001816	0.4521	1.7395	0.0103	0.6888	0.4040	0.7920	0.4500	0.4360	0.0000	0.2270

Abbreviations: ES, enrichment score; NES, normalized ES; NOM, nominal; FDR, false-discovery rate; FWER, family-wise error rate; Tag %, the percentage of gene tags before (for positive ES) of after (for negative ES) the peak in the running enrichment score; gene %, the percentage of genes in the gene list before (for positive ES) of after (for negative ES) the peak in the running enrichment score; GO, gene ontology.

**Table 7:** Main pathways and their genes from the upregulated functional clusters (analyzed with Metascape) in asthmatics versus healthy controls. The genes represented in bold indicate an overlap with the lung cancer dataset.

Pathway Description	List of the Genes Involved
Regulation of cell adhesion	*ANXA1*, ***CD44***, *EGR3*, *FUT3*, *CCN1*, *S100A10*, *CXCR4*, *NR4A3*, ***POSTN***, *MYADM*, *CCDC80*, *S100A8*, *FCGR2A*, *HBB*, *JUN*
Response to activity	*CXCR4*, ***POSTN***, *PPARGC1A*, *G0S2*, ***FOSB***, *PDK4*, *NR4A3*, *PMAIP1*
Embryonic placenta development	*CCN1*, *KRT8*, *SOCS3*, *TM4SF1*, *PSPH*, *ANXA1*, *SERPINB5*, *NR4A3*
Extracellular matrix organization	***CD44***, *CCN1*, *SERPINB5*, ***POSTN***, *CCDC80*, *CXCR4*, *NR4A3*, *SOCS3*
Cell morphogenesis involved in differentiation	*EGR2*, *S100A10*, *CXCR4*, *NR4A3*, ***POSTN***, *MYADM*, *DPYSL3*, ***CD44***
Interferon signaling	***CD44***, *IFIT2*, *SOCS3*
Epithelial cell development	*CXCR4*, *TFCP2L1*, *MYADM*

**Table 8:** Main pathways and their genes from the upregulated functional clusters (analyzed with Metascape) in lung cancer patients versus healthy controls. The genes represented in bold indicate an overlap with the asthma dataset.

Pathway Description	Example of Genes Involved
Signaling by receptor tyrosine kinases	*DUSP6*, *EGFR*, *EGR3*, *ELK1*, *FGFR1*, *FN1*, *FYN*, *GRB2*, *GRB10*, *ID2*, *IGF1R*, *IRSA6A*, *JCAD*, *SNX6*, *PLEK*
Signaling by Rho GTPases	*BRCA1*, *PPP2R2A*, *PPP6C*, *TYMS*, *NSD2*, *WRN*, *ALMS1*, *CDC7*, *RAE1*, *CDC23*, *CCNE2*, *PTTG1*, *KIF23*, *ESPL1*, *RAB1B*, *CEP78*, *NEDD1*
Blood-vessel development	***STAT1***, *EGFR*, ***PPARD***, *PCDC73*, *RAB33B*, *EPPK1*, *FOXP1*, *ANLN*, *CORO1B*, *PLEKHG5*, *EPB41L5*, *ARID5B*, *SYDE1*, *CYGB*, *DNMT1*, *NFATC1*
Regulation of cell projection organization	*FN1*, *FYN*, *GAK*, *GATA3*, ***MYC***, *TGFBR1*, *BCL11A*, *AMIGO2*, *ACTA2*, *NOS1*, *SERPINF2*, *MP14*, *MAP2K5*, *RPS6KA1*, *SLC9A1*, *SPHK1*, *PPM1F*, *ADNP2*, *EXOSC2*
Response to growth factor	*EGFR*, *EGR3*, *FRP4*, *SHC1*, *SPHK1*, *HGS*, *NREP*, *USP15*, ***LUM***, ***POSTN***, *EHD1*, *FERMT2*, *TBC1D7*, *WWOX*, *ERRFI1*, *IL17RD*, *FAM83G*
Extracellular matrix organization	***BCL3***, *BGN*, *BMP1*, *BSG*, *CAPN1*, *CAV1*, *CD36*, *IGFBP4*, *MATN2*, *THBS2*, *TNFAIP6*, *SRPX*, *CILP*, *EDIL3*, *SPON2*, *SPON1*, *MXRA5*, *TSKU*, *CRIM1*, *CTHRC1*, *EMID1*
Response to growth factor	*COL4A2*, *CREBBP*, *DAB2*, *DCN*, *DTYMK*, *DUSP6*, *E2F1*, *EGFR*, *EGR3*, *ERN1*, *FBN1*, *FGFR1*, ***LUM***, ***POSTN***, *EHD1*, *FERMT2*

## Data Availability

All the relevant data are available in the paper and in the Supplementary Materials. The datasets analyzed in the present study can be accessed from https://www.ncbi.nlm.nih.gov/geo last access date was 30 June 2021.

## Supplementary Materials

The following supporting information can be downloaded at: https://www.mdpi.com/article/10.3390/cancers14071663/s1, Supplementary Table S1. List of primers used to validate the genes by RT-qPCR. Supplementary Table S2. List of common pathways identified between GSEA and Metascape analysis in Severe asthma set. Supplementary Table S3. Table for mean expression values and fold change obtained for the eight genes from Microarray analysis for asthmatics, lung cancer, and healthy controls. Supplementary Table S4. Table for gene frequency from GSEA analysis for asthmatics and lung cancer vs. healthy controls. Supplementary Table S5. Table for fold change obtained for the eight genes from RT-qPCR analysis for FFPE from asthmatics (AS), lung cancer (LC), and asthmatics with lung cancer (AC). Supplementary Table S6. Table for log fold change obtained for the eight genes from RT-qPCR analysis for plasma samples asthmatics (AS), lung cancer (LC). Supplementary Table S7. The sample size and log-p rank value from KM Plot. for the eight genes identified from in silico analysis for asthmatics, lung cancer, and healthy controls. Supplementary Table S8. Table for log fold change obtained for the eight genes from RT-qPCR analysis for cell lines from asthmatics (AS) and Lung cancer (LC) patients. Supplementary Table S9. Mortality risk for increased gene expression as per sex, smoking history, cancer stage among lung cancer survival cohorts. Supplementary Figure S1. Boxplots for expression of CD44 probes in healthy, asthmatic, and lung cancers samples from the transcriptomic datasets considered for the study. A. 204489_s_at, B. 204490_s_at, C. 209835_x_at, and D. 212014_x_at. Supplementary Figure S2. Kaplan–Meier survival analysis for four different Affymetrix probes specific for CD44 variants A. 204489_s_at, B. 204490_s_at, C. 209835_x_at, and D. 212014_x_at. Supplementary Figure S3. Kaplan–Meier analysis for the genes showing significantly increased mortality risk for increased expression on overall survival for NSCLC patients selected based on gender, smoking status, and stage. (**A**) BCL3 (Female, nonsmokers, Stage 1); (**B**) BCL3 (Male, Smokers, Stage 1); (**C**) PPARD (Male, smokers, Stage 1) and (**D**) MYC (Male, Smoker, Stage 1). Supplementary Figure S4. Kaplan–Meier analysis for the genes showing significantly reduced mortality risk for increased expression on overall survival for NSCLC patients selected based on gender, smoking status, and stage. (**A**) POSTN (Male, nonsmokers, Stage 1); (**B**) FOSB (Male, nonsmokers, Stage 1); (**C**) FOSB (Male, smokers, Stage 1); (**D**) LUM (Male, nonsmoker, Stage 1); (**E**) LUM (Male, smoker, Stage 1); (**F**) LUM (Female, smoker, Stage 2); and (**G**) LUM (Male, smoker, Stage 2). Supplementary Data S1. Clinical characteristics of the datasets included in the survival analysis (19). Supplementary Data S2. Differentially expressed genes. Supplementary Data S3. Gene Frequency and fold change. Supplementary Data S4. Metascape result for Lung Cancer. Supplementary Data S5. Metascape result for Severe asthma. Supplementary Data S6. CD44 probes list.

## Abbreviations

FFPE	formalin-fixed paraffin-embedded
GINA	The Global Initiative for Asthma
GSEA	gene set enrichment analysis
NSCLC	non-small-cell lung cancer
