# Supplementary material for: In Silico Bioinformatics Followed by Molecular Validation Using Archival FFPE Tissue Biopsies Identifies a Panel of Transcripts Associated with Severe Asthma and Lung Cancer
Source: Cancers (Basel). 2022 Mar 25;14(7):1663. doi: 10.3390/cancers14071663 (PMC8996975; doi:10.3390/cancers14071663)
Supplement: Supplementary file 1 [file cancers-14-01663-s001.zip › Supplementary data 1.pdf]

Supplementary data 1. Clinical characteristics of the datasets included in the survival analysis(19).

Dataset	Platform	Age	Sex (% male)	% of never smokers	Histology (% A/S/L)	Stage (% 1/2/3/4)
GSE4573 (28)	GPL96	67±9.8	63%	3.10%	0/100/0	56/26/18/0
GSE14814 (24)	GPL96	62±8.5	74%	-	31/58/11	50/50/0/0
GSE8894(27)	GPL570	62±10	75%	-	46/54/0	-
GSE19188(25)	GPL570	-	75%	-	49/30/21	-
GSE3141(29)	GPL570	-	-	-	52/48/0	-
GSE31210(21)	GPL570	60±8.1	47%	50%	100/0/0	74/26/0/0
CaArray (26)	GPL96	64±10	51%	14%	-	-
TCGA(22)	GPL3921	66±9.3	67%	7.50%	0/100/0	-
GSE29013(23)	GPL570	64±8.7	69%	3.60%	55/45/0	44/25/31/0
GSE37745(20)	GPL570	64±9.2	55%	-	54/37/12	66/18/14/2
Entire Data Sets		64±10	58%	17.80%	50/45/5	63/27/10/1
