# Supplementary material for: In Silico Bioinformatics Followed by Molecular Validation Using Archival FFPE Tissue Biopsies Identifies a Panel of Transcripts Associated with Severe Asthma and Lung Cancer
Source: Cancers (Basel). 2022 Mar 25;14(7):1663. doi: 10.3390/cancers14071663 (PMC8996975; doi:10.3390/cancers14071663)
Supplement: Supplementary file 1 [file cancers-14-01663-s001.zip › supplementary figs.pdf]

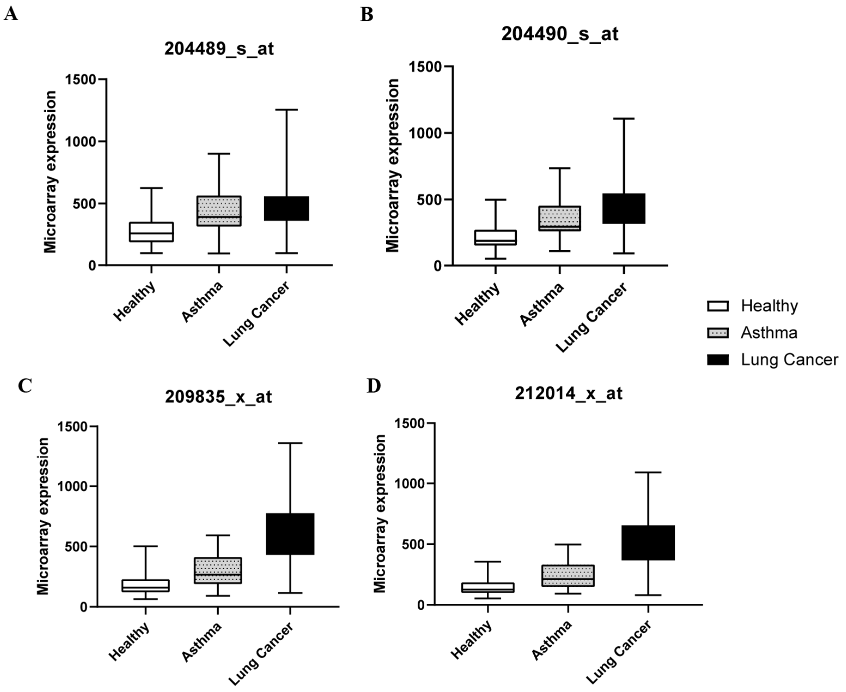

Figure S1. Boxplots for expression of CD44 probes in healthy, asthmatic, and lung cancers samples from the transcriptomic datasets considered for the study. A. 204489_s_at, B. 204490_s_at, C. 209835_x_at, and D. 212014_x_at.

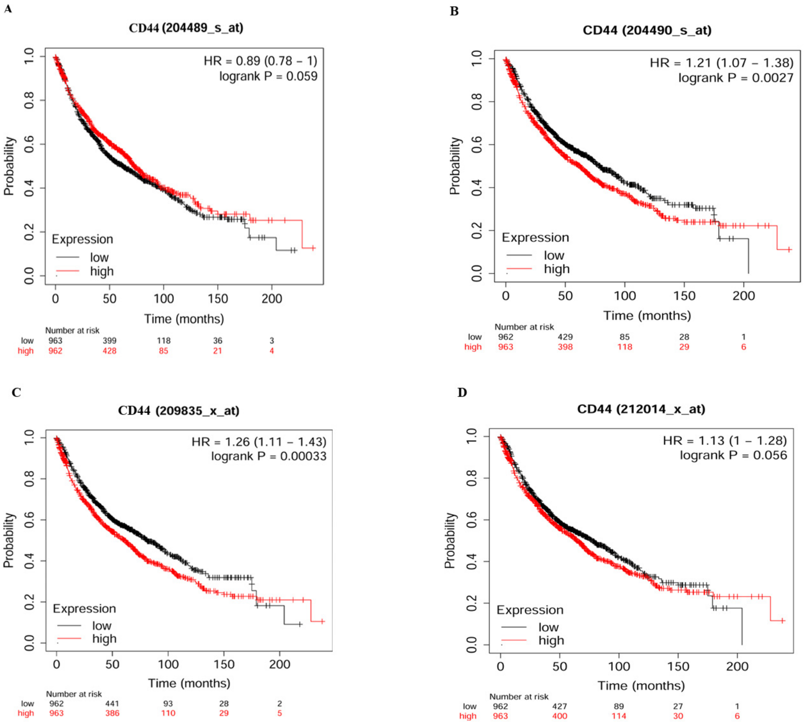

Figure S2. Kaplan–Meier survival analysis for four different Affymetrix probes specific for CD44 variants A. 204489_s_at, B. 204490_s_at, C. 209835_x_at, and D. 212014_x_at.

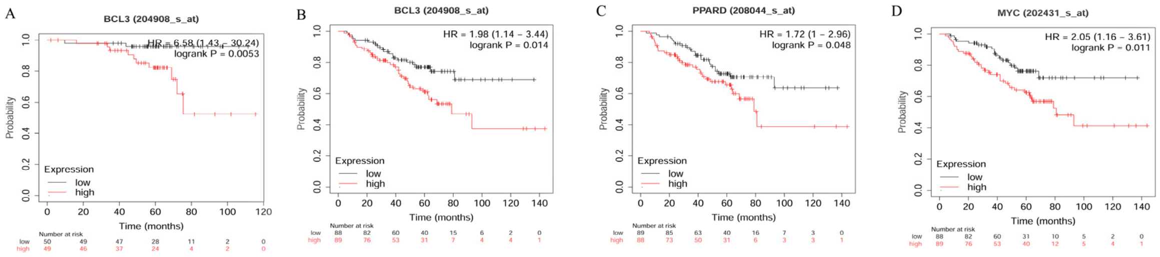

Figure S3. Kaplan– Meier analysis for the genes showing significantly increased mortality risk for increased expression on overall survival for NSCLC patients selected based on gender, smoking status, and stage. (A) BCL3 (Female, nonsmokers, Stage 1); (B) BCL3 (Male, Smokers, Stage 1); (C) PPARD (Male, smokers, Stage 1) and (D) MYC (Male, Smoker, Stage 1).

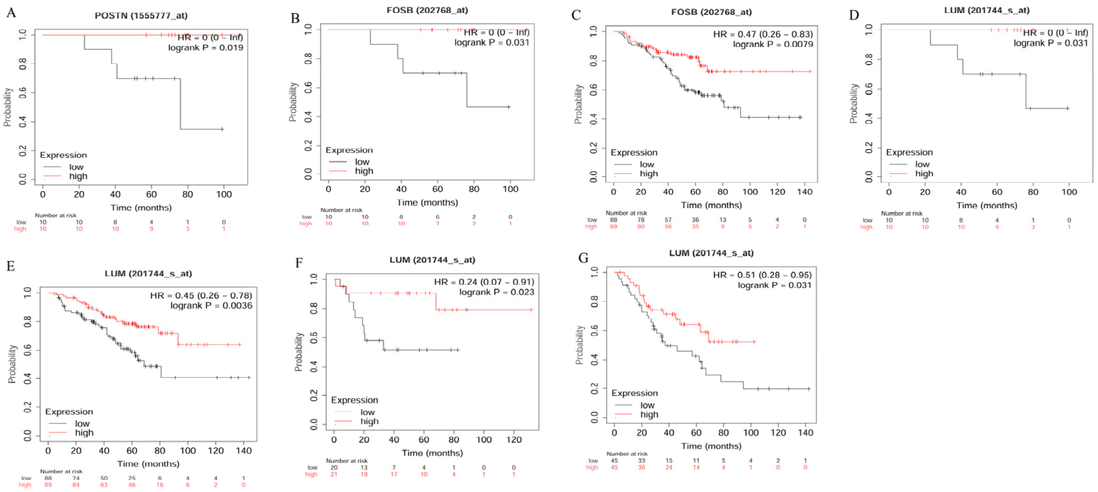

Figure S4. Kaplan–Meier analysis for the genes showing significantly reduced mortality risk for increased expression on overall survival for NSCLC patients selected based on gender, smoking status, and stage. (A) POSTN (Male, nonsmokers, Stage 1); (B) FOSB (Male, nonsmokers, Stage 1); (C) FOSB (Male, smokers, Stage 1); (D) LUM (Male, nonsmoker, Stage 1); (E) LUM (Male, smoker, Stage 1); (F) LUM (Female, smoker, Stage 2); and (G) LUM (Male, smoker, Stage 2).
