# Supplementary material for: In Silico Bioinformatics Followed by Molecular Validation Using Archival FFPE Tissue Biopsies Identifies a Panel of Transcripts Associated with Severe Asthma and Lung Cancer
Source: Cancers (Basel). 2022 Mar 25;14(7):1663. doi: 10.3390/cancers14071663 (PMC8996975; doi:10.3390/cancers14071663)
Supplement: Supplementary file 1 [file cancers-14-01663-s001.zip › Supplementary Tables.pdf]

Supplementary Table S1. List of primers used to validate the genes by RT-qPCR

Gene	Primer	Primer Sequence (5' to 3')	Product length (bp)	NM accession number
POSTN	Forward	TCCTAATGGGGTTGTCACTGT	99	NM_006475.3
	Reverse	TGTGTAAGCACACGGTCAATG		
LUM	Forward	CCTGGAGGTCAATCAACTTGAGA	100	NM_002345.4
	Reverse	GCCATCCAAACGCAAATGCT		
STAT1	Forward	CTTGGATCAGCTGCAGAACT	115	NM_007315.4
	Reverse	AGGGTCATGTTCGTAGGTGT		
CD44	Forward	GGACAAGTTTTGGTGGCACG	100	NM_000610.4
	Reverse	GAATACACCTGCAAAGCGGC		
BCL3	Forward	ACGCAGTGGACATTAAGAGC	118	NM_005178.5
	Reverse	GAGTACATTTGCGCGTTCAC		
FOSB	Forward	GGAGCTGACCGACCGACT	113	NM_006732.3
	Reverse	GCACAAACTCCAGACGTTCCT		
MYC	Forward	CTCCGTCCTCGGATTCTC	120	NM_002467.6
	Reverse	CTTGTTCCTCCTCAGAGT		
PPARD	Forward	CATGACCAAAAAGAAGGCCCG	101	NM_006238.5
	Reverse	CCTTCTCTGCCTGCCACAAT		
18S	Forward	TGACTCAACACGGGAAACC	114	NR_003286
	Reverse	TCGCTCCACCAACTAAGAAC		

Supplementary Table S2. List of common pathways identified between GSEA and Metascape analysis in Severe asthma set

**Common pathways in Asthmatics identified by GSEA and metascape analysis**
positive regulation of cell adhesion
regulation of cell adhesion
reactive oxygen species metabolic process
anion transport
urogenital system development
glycoprotein metabolic process
metal ion homeostasis
nephron development
organic anion transport
gland development
regulation of cell morphogenesis
connective tissue development
sensory system development
epithelial cell proliferation

Supplementary Table S3. Table for mean expression values and fold change obtained for the eight genes from Microarray analysis for Asthmatics, Lung cancer and Healthy controls (Mann–Whitney test, significance P<0.05)

**Gene**	**Asthma (AS) vs Healthy**		**Lung Cancer (LC) vs Healthy**		**AS vs LC**
	**Log Fold Change**	**P value**	**Log Fold Change**	**P value**	**P value**
BCL3	1.961	0.9826	2.683	<0.0001	<0.0001
CD44	2.648	0.0002	3.316	<0.0001	<0.0001
FOSB	2.629	0.0078	2.086	0.6761	0.0004
LUM	-0.010	0.0409	2.733	<0.0001	<0.0001
MYC	2.205	0.6019	2.441	<0.0001	0.0055
PPARD	1.950	0.5218	3.429	<0.0001	<0.0001
POSTN	2.263	0.0010	2.409	<0.0001	0.3132
STAT1	2.504	0.0027	3.202	<0.0001	<0.0001

Supplementary Table S4. Table for gene frequency from GSEA analysis for Asthmatics and Lung cancer Vs Healthy controls

**Gene**	**Frequency from GSEA**	
	**Asthma**	**Lung cancer**
BCL3	9	11
CD44	8	7
FOSB	7	7
LUM	2	2
MYC	6	7
PPARD	16	12
POSTN	6	4
STAT1	6	5

Supplementary Table S5. Table for fold change obtained for the eight genes from RT-qPCR analysis for FFPE from Asthmatics (AS), Lung cancer (LC) and Asthmatics with Lung cancer (AC) (Mann–Whitney test ,significance *P*<0.05)

**Gene**	**Asthma (AS)**	**Asthma with Lung cancer (AC)**	**Lung Cancer (LC)**	**P value**		
	**Log Fold Change**	**Log Fold Change**	**Log Fold Change**	**AS Vs LC**	**AS Vs AC**	**LC Vs AC**
BCL3	0.271	1.475	1.404	0.125	0.922	0.120
CD44	0.276	0.531	1.379	0.023	0.563	0.080
FOSB	0.485	0.314	1.260	0.046	0.642	0.029
LUM	0.284	1.684	1.894	0.008	0.022	0.682
MYC	0.516	0.120	1.431	0.006	0.174	0.001
PPARD	0.210	1.924	1.688	0.026	0.106	0.808
POSTN	0.116	1.221	1.460	0.041	0.101	0.700
STAT1	0.353	1.069	1.493	0.0001	0.004	0.048

Supplementary Table S6. Table for log fold change obtained for the eight genes from RT-qPCR analysis for plasma samples Asthmatics (AS), Lung cancer (LC) (Mann–Whitney test, significance P<0.05)

**Gene**	**Asthma (AS)**	**Lung Cancer (LC)**	**P value**
	**Log fold Change**	**Log fold Change**	
BCL3	-0.103	0.889	0.024
CD44	0.108	1.446	<0.0001
FOSB	-0.295	-1.085	0.104
PPARD	-0.243	2.318	0.000
POSTN	-0.152	1.840	0.000
STAT1	-0.017	1.776	<0.0001

Supplementary Table S7. The sample size and log-p rank value from KM Plot for the eight genes identified from in silico analysis for Asthmatics, Lung cancer and Healthy controls

Gene	Affymetrix ID	Low expression sample size	High expression sample size	Log Rank P value
POSTN	155577_at	577	567	3.7e−06
LUM	201744_s_at	963	962	3.8e−05
BCL3	204907_s_at	964	961	3.6e−05
PPARD	208044_s_at	962	963	4.1e−05
CD44	1557905_s_at	572	572	0.088
MYC	202431_s_at	962	963	5.8e−06
FOSB	202768_at	966	959	0.0013
STAT1	209969_s_at	962	963	0.84

Supplementary Table S8. Table for log fold change obtained for the eight genes from RT-qPCR analysis for cell lines from Asthmatics (AS) and Lung cancer (LC) patients

Gene	Asthmatic cells			Lung cancer cells		
	DHBE	S13	S14	A549	SK-Lu-1	Calu3
BCL3	0.010	-0.094	-0.579	0.402	0.296	0.308
CD44	-0.057	-0.096	-0.142	0.374	0.274	0.192
FOSB	0.335	-0.005	-0.330	1.761	1.229	0.014
PPARD	0.004	-0.247	-0.009	0.276	0.323	0.332
POSTN	0.021	0.718	-0.718	-0.720	-0.358	-2.104
STAT1	0.010	-0.067	0.033	0.313	0.207	0.141

Supplementary Table S9: Mortality Risk for Increased gene expression as per sex, smoking history, cancer stage among lung cancer survival cohorts.

**Probe ID**	**Gene Symbol**	**Sex**	**smoking**	**Cancer Stage**	**Log rank P-Value**
Increased Risk					
204908_s_at	BCL3	Female	Never Smoked	Stage 1	0.0053
204908_s_at	BCL3	Male	Smoker	Stage 1	0.014
208044_s_at	PPARD	Male	Smoker	Stage 1	0.048
202431_s_at	MYC	Male	Smoker	Stage 1	0.011
Decreased Risk					
1555777_at	POSTN	Male	Never Smoked	Stage 1	0.019
202768_at	FOSB	Male	Never Smoked	Stage 1	0.031
202768_at	FOSB	Male	Smoker	Stage 1	0.0079
201744_s_at	LUM	Male	Never Smoked	Stage 1	0.031
201744_s_at	LUM	Male	Smoker	Stage 1	0.0036
201744_s_at	LUM	Female	Smoker	Stage 2	0.023
201744_s_at	LUM	Male	Smoker	Stage 2	0.031
